# A computational framework for discovering digital biomarkers of glycemic control

**DOI:** 10.1038/s41746-022-00656-z

**Published:** 2022-08-08

**Authors:** Abigail Bartolome, Temiloluwa Prioleau

**Affiliations:** Dartmouth College, Computer Science, Hanover, NH 03755 USA

**Keywords:** Biomedical engineering, Type 1 diabetes

## Abstract

Digital biomarkers can radically transform the standard of care for chronic conditions that are complex to manage. In this work, we propose a scalable computational framework for discovering digital biomarkers of glycemic control. As a feasibility study, we leveraged over 79,000 days of digital data to define objective features, model the impact of each feature, classify glycemic control, and identify the most impactful digital biomarkers. Our research shows that glycemic control varies by age group, and was worse in the youngest population of subjects between the ages of 2–14. In addition, digital biomarkers like prior-day time above range and prior-day time in range, as well as total daily bolus and total daily basal were most predictive of impending glycemic control. With a combination of the top-ranked digital biomarkers, we achieved an average F1 score of 82.4% and 89.7% for classifying next-day glycemic control across two unique datasets.

## Introduction

Maintaining good glycemic control is the primary and daily objective for persons with diabetes. Tangibly, this equates to managing several factors that can affect blood glucose such as food, medication, activity, and much more^1–4^. Research shows that persons with diabetes make over 100 health-related decisions and spend an average of 58 mins per day on self-care related to their condition^5,6^. This daily effort is critical to minimize the occurrence of adverse glycemic events (i.e., hypoglycemia and hyperglycemia). In addition, maintaining good glycemic control is imperative to delay and possibly avoid long-term diabetes complications like kidney failure and peripheral arterial disease^7,8^. Hence, the use of digital tools like continuous glucose monitors (CGMs) and insulin pumps is growing to support the complex task of managing diabetes^9–12^.

CGMs and insulin pumps are clinically-validated minimally-invasive wearable systems that enable continuous monitoring of blood glucose concentrations, and semi-automatic administration of insulin for the goal of maintaining normoglycemia (i.e., normal blood glucose levels). The benefits of using these wearable medical devices in diabetes care is two-fold. First, CGMs and insulin pumps enable real-time awareness of blood glucose levels and in-the-moment response to current or impending adverse glycemic events^13,14^. Second, CGMs and insulin pumps provide a rich data source for retrospective learning to guide future management strategy. However, research shows that these data sources are significantly underutilized^15,16^. Given the growing national and global burden of diabetes^17–20^, there are unique and untapped opportunities for intelligent computational methods that can leverage the vast amount of digital data from CGMs, insulin pumps, and other devices to increase our understanding of the association and impact of individualized daily factors on diabetes outcomes.

The importance of digital biomarkers in the health domain is irrefutable^21–28^. Digital biomarkers refer to objective, quantifiable, physiological, and behavioral measures that are collected by means of digital devices, such as wearable devices, for the purpose of explaining, influencing, or predicting health outcomes^21,29,30^. However, there is limited research on digital biomarkers relevant to chronic conditions like diabetes. Despite the deluge of digital data that is collected from routine use of wearable medical devices like CGMs and insulin pumps, there are many unanswered questions about quantifiable measures that are associated with and/or predictive of daily glycemic control.

**Fig. 1:**
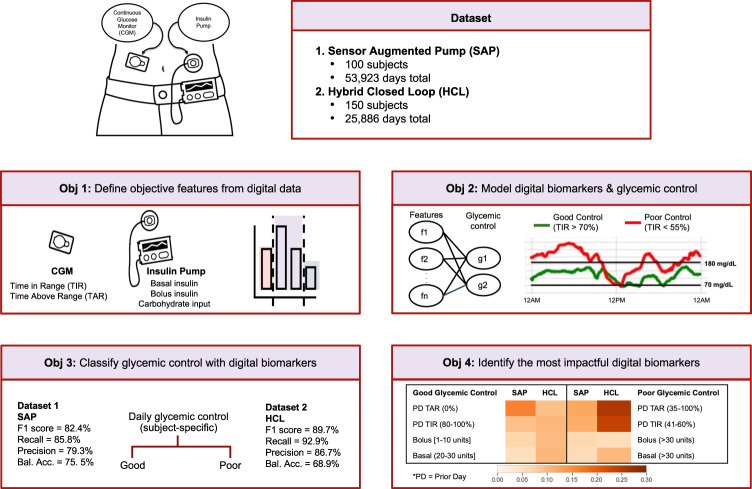
Research overview. This study leverages over 79,000 days of CGM and insulin pump data from 250 subjects for four objectives: 1) define objective features from digital data, 2) model digital biomarkers and glycemic control, 3) classify glycemic control with digital biomarkers, 4) identify the most impactful digital biomarkers associated with good and poor glycemic control and consistent across two distinct datasets, i.e., sensor augmented pump (SAP) and hybrid closed loop (HCL).

To support an increased use of routinely collected data from a myriad of digital devices and improve our understanding of complex factors that affect diabetes, this research makes important contributions by proposing and evaluating a scalable computational framework for discovering digital biomarkers of glycemic control. As a feasibility study, we leverage over 79,000 days of digital data from CGMs and insulin pump to: 1) define objective features from digital data, 2) model digital biomarkers and glycemic control, 3) classify glycemic control with digital biomarkers, and 4) identify the most impactful digital biomarkers for impending glycemic control (see Fig. 1). Our dataset is orders of magnitude larger than related research in the field^2,27,31–34^. In addition, our proposed framework can be easily expanded to integrate and incorporate continuous streams of other relevant data sources, such as from wearable activity trackers like Fitbit, to discover the impact of daily activity level, sleep, stress, circadian rhythm, and much more, on glycemic control. Our proposed method is intuitive and it provides interpretable and actionable insights that can further guide daily management strategies to improve diabetes outcomes.

## Results

### Defining objective features from digital data

In this work, our digital data comes from CGMs and insulin pumps used by 250 subjects with diabetes, primarily type 1 diabetes (ages 2–76 years, diabetes duration is between 0 and 60 years); see Table 2 in the Methods section for more details. This dataset represents two unique populations including 100 subjects that use sensor augmented pump (SAP) therapy and 150 subjects that use a hybrid closed loop (HCL) system for daily management of their diabetes. In SAP therapy, the patients’ insulin pump is in communication with their CGM, thus the pump can suspend insulin delivery when blood glucose readings from the CGM are low (or getting low) to reduce hypoglycemia^35^. Meanwhile, in the HCL case, the patients’ CGM plus insulin pump system includes a more sophisticated algorithm that automatically adjusts primarily basal insulin (i.e., small amounts of background insulin) from the pump in response to blood glucose readings from the CGM^36,37^. However, in both cases (SAP and HCL), a person with diabetes is expected to “announce meals" by entering the estimated carbohydrate amount into their insulin pump to determine and administer bolus insulin (i.e., larger amounts of fast-acting insulin) needed to account for food consumption^38,39^. Thus, from both SAP and HCL populations, the diabetes-device dataset includes blood glucose data, recorded every ≈ 5 min from CGMs, as well as amounts and timestamps of bolus insulin doses, basal insulin doses, and carbohydrate amount entries from the insulin pumps.

**Fig. 2:**
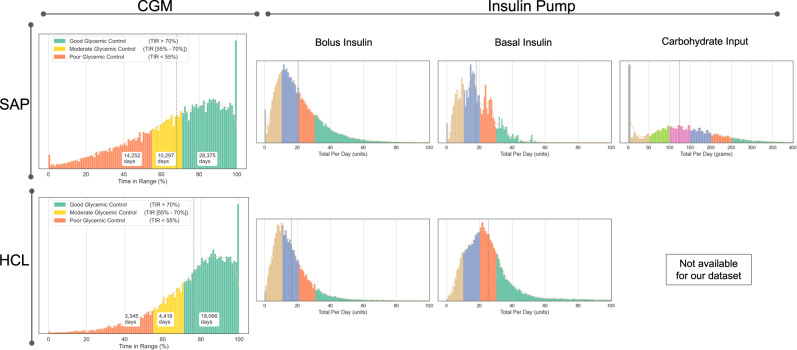
Overview and distribution of digital data. This figure shows the distributions across subjects in the SAP and HCL population for time in range from the CGMs and total bolus, basal and carbohydrate input per day from the insulin pumps.

For the objective of leveraging digital data to discover biomarkers of glycemic control (i.e., the outcome variable), we defined features using domain knowledge and data-driven insights^2,14,40–43^. We define good glycemic control based on the clinical target for persons with type 1 diabetes, which is to maintain blood glucose between 70 and 180 mg/dL for greater than 70% of a 24-h day^40^. Using the aforementioned target as an anchor, we define poor glycemic control as days when the time in range (TIR) is less than 55% in a 24-h day. Our definition is based on the intuition that if TIR greater than 70% is recognized clinically as good glycemic control, then TIR equal to 68% or 65% should not arbitrarily be classified as poor glycemic control. Thus, we consider the TIR between 55% and 70% as moderate glycemic control (i.e., not good and not poor). Additionally, we defined descriptive features for the input data (i.e., retrospective CGM data and insulin pump data). Based on the distribution of each data stream shown in Fig. 2, we identified patterns of behaviors that might impact impending glycemic control by stratifying each data stream into unique ranges. For example, total daily bolus insulin was described with one of four features: no entry, 1–10 units, 10–20 units, 20–30 units, and greater than 30 units. Large daily bolus insulin amounts (e.g., >30 units/day) could be representative of large meals, meanwhile low daily carbohydrate inputs (e.g., 5–50g per day) could be representative of a low-carb diet^43,44^ or missed meal announcements^45–47^. In total, our input variable feature set comprising 26 features from five categories included two from the prior-day CGM data and three from the current-day insulin pump data. Supplementary Table 1 shows the full set of objective features.

The analysis in this step highlighted some key observations. From Fig. 2 and Table 1, we observe that the distribution of CGM data from the HCL population is more asymmetric and negatively skewed with a thinner tail on the left (mean TIR = 76.3%, median TIR = 80.4%) compared to the SAP population (mean TIR = 68.0%, median TIR = 72.2%). This indicates that there were more occurrence of high TIR (i.e., good glycemic control) in the HCL population compared to that of the SAP population. Prior work has found similar results and supports that patients with HCL systems often achieve better glycemic control and more time with their blood glucose levels in the target range of 70–180 mg/dL than patients with SAP therapy^39^. Better glycemic control in the HCL population is likely due to the automatic and adjustable nature of basal insulin delivery, in contrast to the generally fixed nature of basal insulin delivery in the SAP population. In addition, from Fig. 2 and Table 1 respectively, we can also observe a smaller (or thinner) tail on the bolus insulin distribution with a slightly lower mean of 16.3 units per day in the HCL population compared to the SAP population with a mean of 20.4 units per day. This represents fewer occurrences of large daily bolus insulin in the HCL population compared to the SAP population. Conversely, we observe a wider tail on the basal insulin distribution and a mean of 25.5 units per day in the HCL population which represents more occurrences of larger daily basal insulin compared to the SAP population with a mean of 18.3 units per day. Based on the observed differences, we modeled the impact of the defined digital biomarkers on daily glycemic control to: 1) investigate the ability to classify good and poor glycemic control using a combination of those features, and 2) identify the most impactful digital biomarkers across both the SAP and HCL datasets.

**Table 1:** Summary metrics for digital biomarkers in the SAP (*n* = 100) and HCL (*n* = 150) population.

Dataset	Digital biomarkers	Mean	Median	Std Dev	Range	Skewness	Kurtosis
SAP	Time in range (%)	68.0	72.2	23.2	0–100	−0.71	−0.20
	Time above range (%)	28.1	22.9	24.4	0–100	0.79	−0.18
	Bolus insulin (units per day)	20.4	17.2	14.0	0–163	1.53	4.05
	Basal insulin (units per day)	18.3	16.6	10.3	0–98	0.88	1.36
	Carb. input (grams per day)	124.3	120.0	88.6	0–598	0.49	0.10
HCL	Time in range (%)	76.3	80.4	18.5	0–100	−1.12	1.23
	Time above range (%)	20.1	14.9	19.4	0–100	1.23	1.33
	Bolus insulin (units per day)	16.3	13.7	12.0	0–244	2.78	18.62
	Basal insulin (units per day)	25.5	22.6	17.0	0–185	2.71	11.75

### Modeling digital biomarkers and glycemic control

There are well-known behavioral and lifestyle factors that affect glycemic control^42–44,48–54^. However, there is little research on understanding how trends and individualized patterns amongst lifestyle factors are associated with good and poor glycemic control. This knowledge can be very beneficial for patients with diabetes and can provide actionable insights on what exactly a person can change to achieve better glycemic control on a daily basis^2,16,27^. To address this critical need, we modeled the impact of digital biomarkers from retrospective CGM data (i.e., using prior data only) and insulin pump data on the present-day to understand daily glycemic control. Our approach uses a form of topic modeling, particularly supervised latent Dirichlet allocation (sLDA)^55^, which was originally proposed for text mining, but has since been extended to other data types and applications^56–58^. Unlike the standard LDA with documents and words, our approach uses data streams from the CGM (using prior data only) and insulin pump as “documents” and patterns of behaviors (e.g., large daily bolus insulin) as “words” (or features) that are then mapped to impending glycemic control as the outcome variable. Using our defined digital biomarkers (*n* = 26) from the feature categories of prior-day glycemic control (quantified using prior-day TIR and prior-day TAR), and insulin pump features (quantified using daily bolus insulin, daily basal insulin, and daily carbohydrate input), we modeled good (TIR > 70%) and poor (TIR < 55%) glycemic control. Since sLDA is an extension of the unsupervised latent Dirichlet allocation (LDA)^59^, which employs a random seed for modeling, there can be variation in the results obtained from multiple trials. Hence, we repeated our modeling *k* = 3 times to ensure stable findings on digital biomarkers that impact daily glycemic control.

**Fig. 3:**
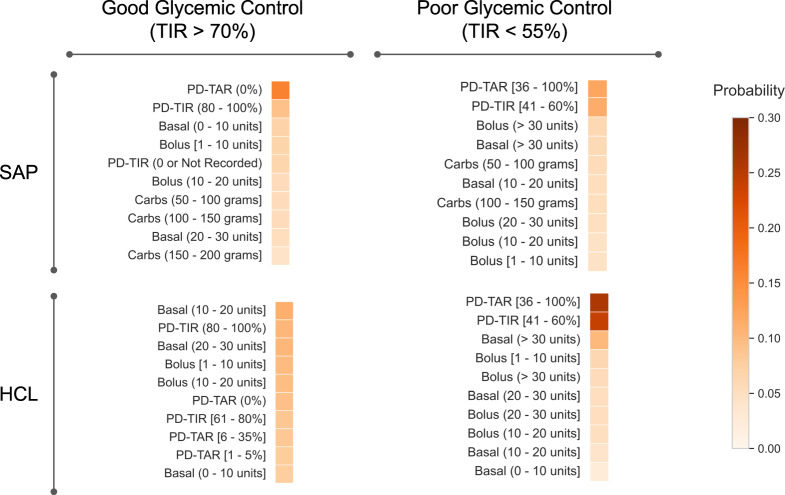
Top 10 digital biomarkers associated with good and poor glycemic control for the SAP and HCL datasets. This figure shows the probabilities obtained from modeling the defined objective features and daily glycemic control using sLDA.

Figure 3 shows the top 10 digital biomarkers associated with good and poor glycemic control in the SAP and HCL populations. Supplementary Table 2 shows similar results from two additional runs with different random seeds. We observe that a combination of prior-day TIR between 80 and 100% and smaller daily basal and bolus insulin amounts, generally less than 30 units per day, are amongst the top digital biomarkers with the highest probabilities associated with good glycemic control. Meanwhile, a combination of prior-day time above range (TAR) between 36 and 100%, prior-day TIR between 41 and 60%, and larger daily bolus insulin greater than 30 units per day are amongst the top digital biomarkers with the highest probabilities associated with poor glycemic control. These results were found to be stable across repeated evaluations and across both the SAP and HCL dataset.

### Classifying glycemic control with digital biomarkers

**Fig. 4:**
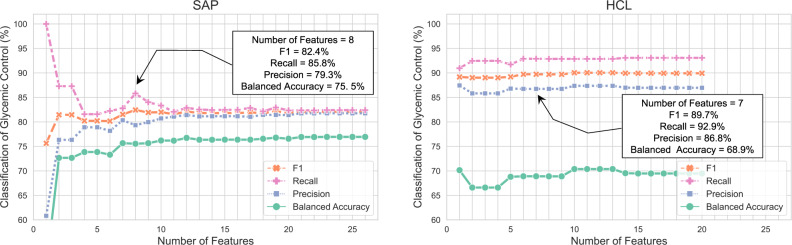
Feature importance analysis for classification of glycemic control. The classification results for the SAP and HCL dataset plateaued after inclusion of the top 7–8 objective features. This figure shows the results obtained with a logistic regression classifier.

Predicting glycemic control with digital biomarkers is useful to further evaluate the importance and relevance of the defined features. This knowledge can also provide a greater understanding of glycemic control and actionable insights for persons with diabetes who use continuous digital devices for management of their condition. Toward this goal, we started with the single top-ranked feature in each dataset, as shown in Fig. 3 and Supplementary Table 3, then incrementally added one feature with the next highest probability for iterative classification of good and poor glycemic control. As shown in Fig. 2, our dataset includes a total of 42,817 and 20,879 days of good plus poor glycemic control for the SAP and HCL populations, respectively. We evaluated several classifiers using an 80/20 train/test split for classification of good versus poor glycemic control and observed that the results plateaued after inclusion of 7–8 features for both datasets. Figure 4 shows the results of this analysis for the logistic regression classifier while Supplementary Table 5 shows the results with other classifiers including support vector machine, decision tree, and a linear discriminant analysis classifier. For classification of glycemic control, we achieved an F1 score of 82.4% and balanced accuracy of 75.5% (recall = 85.8% and precision = 79.3%) for the SAP dataset using the top 8 features. Meanwhile, we achieved an F1 score of 89.7% and balanced accuracy of 68.9% (recall = 92.9% and precision = 86.8%) for the HCL dataset using the top 7 features. Supplementary Fig. 1 shows the confusion matrix for results obtained with the logistic regression classifier. From Fig. 4, we note that the classification results for the HCL dataset was relatively stable (i.e., <5% difference) independent of the number of features used. This is likely due to significant class imbalance in the HCL dataset with approximately 4-times more occurrences of good glycemic control (TIR > 70%) as opposed to poor glycemic control (TIR < 55%) - see Fig. 2. The presence of more days with good glycemic control, which equates to class imbalance, in the HCL dataset is representative of real use of this system for diabetes management. Thus, we calculated and reported the balanced accuracy^60^ in our analysis. Our results indicate that the top 7–8 features shown in Fig. 3 and Supplementary Table 3 are useful digital biomarkers that impact glycemic control. Some of these features are modifiable behaviors and thus provide actionable insights on what a person with diabetes can change to achieve better glycemic control.

### Identifying the most impactful digital biomarkers that impact glycemic control

Similar to traditional biomarkers, digital biomarkers are objective measures that can be used to explain, influence, or predict health-related outcomes^30^. However, unlike traditional biomarkers that provide a “snapshot view" based on limited measurements collected over time, digital biomarkers are often derived from longitudinal and continuous measurements, and thus can capture dynamic changes in health and related outcomes^21–26,30,61^. Consequently, an important but critical part of the process of developing digital biomarkers is sifting through large volumes of often heterogeneous data sources to identify pertinent features or measures. Through our systematic approach of defining objective features from digital data (particularly CGM and insulin pump data), modeling digital biomarkers and glycemic control, and classifying glycemic control with digital biomarkers, we learned that prior-day time above range (PD-TAR) and prior-day time in range (PD-TIR) are most predictive digital biomarkers for both good and poor glycemic control on a given day. As shown in Fig. 3 and in Supplementary Tables 2 and 3, good glycemic control with PD-TAR equal to 0% and PD-TIR in the range of 80–100% was most predictive of good glycemic control for the next day. Conversely, poor glycemic control with PD-TAR in the range of 36–100% and PD-TIR in the range of 41–60% was most predictive of poor glycemic control for the following day. The next most impactful digital biomarker observed through multiple iterations of modeling was related to the daily total units of basal and bolus insulin. More specifically, a daily total of less than 30 units of basal and bolus insulin was often associated with good glycemic control while a daily total of greater than 30 units was often associated with poor glycemic control. Although carbohydrate input data was only present for the SAP dataset, our results show daily total of carbs in the range of 50–100 g and 100–150 g was often associated with poor glycemic control. The aforementioned digital biomarkers were consistently amongst the top 5 features associated with good or poor glycemic control. Thus, a combination of these features proved to be particularly useful for predicting glycemic control.

## Discussion

Diabetes is a prevalent chronic condition that is increasingly affecting more children, youths, and adults, both nationally and globally^17–20^. Management of this life-long condition is a daily challenge that requires maintaining good glycemic control to reduce the occurrence of short- and long-term complications^7^. However, research shows that less than one-third of people living with type 1 diabetes are consistently achieving the recommended goals for glycemic control^10^. As a result, digital technology like CGMs, insulin pumps, and automated insulin delivery systems are increasingly prescribed and used by persons with diabetes to reduce the occurrence of adverse health events and the burden of management^9,10^. The routine use of ubiquitous digital devices provides unique opportunities to gain a greater understanding of the condition, the impact of behavioral and lifestyle choices, and develop digital biomarkers from longitudinal and continuous measurements to further support patients, families, and care teams with improving management.

In this work, we present a scalable computational framework for leveraging digital data from wearable devices to discover and identify digital biomarkers for understanding daily glycemic control. Our proposed framework includes defining objective features from digital data, modeling the digital features with clinically-validated metrics for glycemic control, and investigating the relative importance of the defined features for predicting glycemic control. From implementing and evaluating the proposed framework on large SAP and HCL datasets, our results showed that features from the CGM, particularly prior-day TIR and prior-day TAR, are the most predictive digital biomarkers for good and poor glycemic control. We also found that insulin-related features, particularly total daily basal insulin and total daily bolus insulin, are relevant digital biomarkers of glycemic control. For classification of glycemic control, we found that the top-ranked 7–8 digital biomarkers shown in Fig. 3 were sufficient for prediction and achieved an F1 score of 82.4% in the SAP dataset and 89.7% in the HCL dataset. A natural extension of this work is to leverage the proposed framework for discovering more digital biomarkers of daily glycemic control by studying the relationship and impact of other relevant factors like activity level, sleep, stress, circadian rhythm, and more, many of which can be monitored with consumer-grade wearable devices like Fitbit^62,63^.

**Fig. 5:**
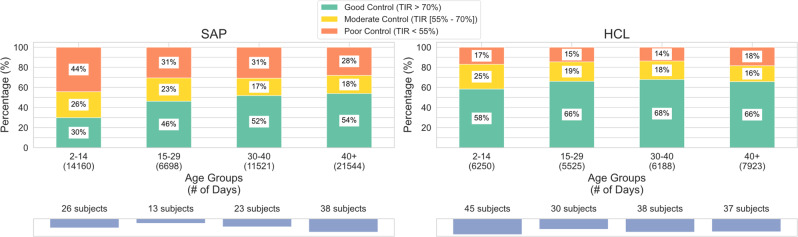
Glycemic control by age group. Glycemic control improved with age in the SAP population and was worse in the youngest group of subjects between the ages 2–14 across both the SAP and HCL populations.

Compared to prior and related work, our research is unique for three reasons. First, we propose and evaluate a scalable computational framework for leveraging large amounts of continuously sensed and grossly underutilized digital data to discover biomarkers of daily glycemic control. This work sets a precedence for use of routinely collected digital data to understand the complex combination of factors that impact personalized diabetes management. Second, this research leverages a large dataset with 250 subjects and over 79,000 days of data from two unique patient populations (i.e., SAP and HCL) to evaluate the proposed computational framework. Our dataset is orders of magnitude larger than those used in related studies^2,27,31–34^. In addition, unlike prior work on digital biomarkers in various health domains^23,26,56,57^, our study does not rely on self-reported data which is often biased, instead we use data from clinically-validated medical devices. Third, since our dataset was collected through routine use of wearable devices for daily management of diabetes, this observational data can reveal behaviors/factors in the natural environment that are not influenced or altered as a result of enrolling in a research study^64^. For example, exploratory analysis on the distributions of CGM data in different populations showed more occurrence of high TIR (i.e., good glycemic control) for patients with HCL systems compared to patients with SAP therapy (see Fig. 2). This finding aligns with results from prior literature^39^. Additionally, we explored differences in glycemic control across age groups by stratifying subjects in this study into four groups (i.e., ages 2–14, 15–29, 30–40, and 40+). Figure 5 shows that glycemic control improved with age and was worse in the youngest group of subjects between the ages of 2–14. This trend is particularly evident in the SAP population and less prevalent in the HCL population. It is worth noting that our proposed computational framework, modeling, and results are interpretable and provide actionable insights. For example, our research showed that prior-day glycemic control is the most predictive digital biomarker associated with present-day glycemic control. This finding is intuitive, interpretable, and in alignment with results from recent research involving patients with type 1 diabetes who use a closed-loop control system (as opposed to SAP or HCL as in this study) for daily management of their condition^65^.

However, despite the contributions of this research, there are some limitations that are worth noting. One of such limitation relates to representation in our study cohort. More specifically, it is well-known that individuals who have access to digital devices may not represent the full spectrum of the population of interest, especially in health-related conditions^64^. For example, Foster et al. found that racial differences and disparities exist in the use of technology for health management, including the use of insulin pumps and CGMs for diabetes management^10^. Although the race and ethnic distribution of subjects in our study is unknown, it is important to recognize that there may be some underlying disparities. In addition, adherence of personal health devices is often varied because people do not always use or wear these devices; this is also the case for wearable medical devices like those used in this study^66,67^. In addition, our dataset has less than 10% of missing data, hence it may represent more motivated patients who are already interested in leveraging digital data to improve management of their diabetes. Lastly, it is well-known that differences in the type and quality of carbohydrates (and other macronutrients) can affect blood glucose differently. However, given that our dataset comes directly from CGMs and insulin pumps, it does not include information on the type and quality of meals.

Building on this research, there are several important avenues to be explored in future work. For example, this study only included digital data from CGMs and insulin pumps, thus, a compelling future direction is to integrate other streams of data that capture relevant behaviors that affect glycemic control such as activity, sleep, stress, etc. This data fusion can be achieved by leveraging wearable activity trackers in addition to CGMs and insulin pumps. Another future direction includes broadening representation in the patient cohort and conducting a prospective study to further validate the insights learned from this research. Finally, it is important to evaluate how knowledge from this and similar research can be useful to the larger population of people and patients who may not use continuous monitoring devices for daily management of their health. This includes the vast population of patients with type 2 diabetes who use glucometers for point-in-time measurements of the blood glucose and insulin pens for multiple daily injections.

## Methods

### Dataset

Table 2 summarizes our dataset which includes 250 subjects with diabetes, primarily type 1 diabetes (age = 2–76 years, time with diabetes = 0–60 years). This dataset is from the Tidepool Big Data Donation Project^68^. It was licensed and approved for use in this study by the Committee for Protection of Human Subjects at Dartmouth College. The experiments and methods are carried out in accordance with all ethical regulations and the license agreement. In addition, all subjects provided informed consent for use of their diabetes device data. The dataset represents two populations, namely, patients who use SAP therapy^35,69^ (*n* = 100) and patients who use HCL^36,37,39^ (*n* = 150) for daily management of their diabetes. On average, there was 522 days (≈1 year and 5 months) of data per subject in the SAP population and 172 days (≈5–6 months) of data per subject in the HCL population. In both populations, patients use CGMs, which are clinically-validated and minimally-invasive wearable devices for continuous monitoring of their blood glucose^14,40^. CGMs on the market today (e.g., those by Dexcom^70^, Medtronic^71^, and Abbott^72^) record 1 blood glucose sample every 1–5 min. Thus, our dataset includes an average of 144,162 blood glucose samples per subject (total = 14.4 million samples across 100 subjects) in the SAP population and an average of 47,875 blood glucose samples per subject (total = 7.1 million samples across 150 subjects) in the HCL population.

**Table 2:** Summary of study dataset (*n* = 250 subjects with diabetes) including descriptive characteristics of the sensor augmented (SAP) therapy and hybrid closed loop (HCL) populations.

		SAP		HCL	
	*Note: NR = Not Reported	Mean ± SD	Range	Mean ± SD	Range
Subject	Total no. of subjects	100		150	
	Male/Female/NR	23/13/64		47/66/37	
	T1D/T2D/NR	41/0/59		142/0/ 8	
	Age of subjects	34.38 ± 20.57	2–76	28.56 ± 16.19	3–75
	Yrs. w/ diabetes	18.41 ± 16.68	0–60	12.07 ± 15.32	0–50
	No. of days/subject	521.73 ± 179.71	306–1087	171.7 ± 103.58	94–683
CGM	Total BG samples	14,416,242		7,181,253	
	Samples/subject	144,162 ± 49,346	85,251–294,476	47,875 ± 28,846	25,861–194,858
Insulin Pump	Total bolus doses	383,868		200,888	
	Bolus doses/subject	3838.68 ± 1948.48	1227–10,857	1339 ± 841	451–5467
	Total carb-inputs	208,582		Not in Dataset (N/A)	
	Carb-inputs/subject	2085.82 ± 1529.87	0–9766	(N/A)	(N/A)
	Total 24-h basal doses	51,231		25,879	
	24-h basal/subject	512.31 ± 193.78	224–1139	172.53 ± 104.59	94–699

In addition, all subjects also used insulin pumps for daily management of their diabetes. Hence, the insulin pump data includes basal, bolus, and meal-related entries (or carb-inputs) for each subject. Basal insulin^38^ refers to small amounts of background insulin infused from the pump either on a programmed schedule as in the SAP population or automatically adjusted by an algorithm as in the HCL population. Meanwhile, bolus insulin refers to larger amounts of rapid-acting insulin required to cover meals and correct for high blood glucose levels^38^. Our dataset includes an average of 3838 bolus insulin doses per subject and 2085 carb-inputs per subjects (total = 383,868 insulin doses and 208,582 carb inputs across 100 subjects) in the SAP population, and 1339 bolus insulin doses per subject and unknown carb-input data (total = 200,888 bolus insulin doses across 150 subjects) in the HCL population. Since basal insulin is administered at an infusion rate (i.e., units per hour) which can vary for different time periods in the day, we calculated basal insulin amounts for 24-h periods in our dataset, thus the 24-h basal per subject in both dataset is approximately equal to the number of days of data per subject. This rich dataset represents an example of the prevalence of routinely collected digital data that is grossly underutilized in healthcare. Our research provides a scalable computational framework to facilitate improved use of such data to understand the complex combination of factors that affect diabetes management and discover digital biomarkers of glycemic control.

### Defining objective features from digital data

In this work, we use CGM and insulin pump data described in Table 2 to define objective features for modeling and predicting daily glycemic control. Our feature set comprises of digital biomarkers from five categories outlined in Table 1, including two from prior-day CGM (i.e., time in range and time above range) and three from the current-day insulin pump (i.e., total bolus, basal, and carb inputs per day). Each feature category was split into 4–7 unique ranges based on the data distribution across samples from all subjects, thus yielding discrete features like prior-day TIR between 41 and 60% or total bolus insulin per day between 10 and 20 units. Supplementary Table 1 outlines all 26 features defined in this work. Prior-day CGM features were based on domain-knowledge which supports the use of time in different glycemic ranges as a validated and clinically-relevant metric for accessing diabetes management^40,73,74^. More specifically, TIR - defined as the percentage of blood glucose readings from a CGM between 70 and 180 mg/dL, and TAR - defined as the percentage of blood glucose readings greater than 180 mg/dL^40^, were used. Both metrics were calculated on a daily basis (i.e., within a 24-h window from 12AM - 11:59PM). In this study, we did not include time below range (TBR) as a separate feature because it is indirectly accounted for by the inclusion of TIR and TAR. In addition to the aforementioned retrospective data from the CGMs, we also included insulin pump data from the current day of interest for the goal of learning data-driven insights that can inform in-the-moment decision making for persons with diabetes. All features were defined to quantify behaviors/factors such as adherence to self-management tasks like meal announcements^45^ or bolus insulin dosing^46^ and dietary habits like conforming to low-carb or high-carb diets^48,75^, each of which can have an impact on daily glycemic control and long-term diabetes outcomes.

### Modeling digital biomarkers and glycemic control

To further understand how our defined features are associated with impending glycemic control, we used sLDA^55^, a form of probabilistic topic modeling for analysis^76^. Our approach builds on prior research that has shown the applicability of LDA and sLDA for modeling different health-relevant data types^56–58^. However, in this study, sLDA was used to infer latent features (i.e., input variables) that are predictive of good and poor glycemic control (i.e, the response variable). The clinically-validated metric, TIR, was used to stratify both good and poor glycemic control^40,73,74^. More specifically, the clinical target of TIR > 70% was classified as good glycemic control, while TIR < 55% was classified as poor glycemic control (see Fig. 2). In the SAP population, there were a total of 17,915 days with poor glycemic control and 24,902 days with good glycemic control. Conversely, in the HCL population, there were 4178 days with poor glycemic control and 16,701 days with good glycemic control. Every digital feature and the associated response variable was calculated on a daily basis. There were a total of 26 features from the SAP dataset and 20 features from the HCL dataset which was missing the carbohydrate input data stream (see Supplementary Tables 1 and 3). The tomotopy toolkit^77^ in Python was used to implement sLDA for modeling. Following this, term-frequency inverse document frequency (tf-idf) was used to weigh each input to adjust for the fact that some features appear more frequently in the dataset^78^. Finally, as a validation step, sLDA was run multiple times (*k* = 3) with different random seeds to ensure stable and reproducible findings on the ranking and associations between digital biomarkers and glycemic control (see Fig. 3 and Supplementary Table 2).

### Classifying glycemic control with the most impactful digital biomarkers

To further investigate the potential of the defined objective features as digital biomarkers of glycemic control, we leveraged these features for classification. More specifically, we partitioned both datasets (i.e., SAP & HCL) into a training and test set using an 80/20 split (see Supplementary Table 4 for a breakdown of the number of samples in each partition). Following this, we evaluated and compared the classification results using 4 interpretable and explainable classic models^79,80^, namely, logistic regression, support vector machine with a radial basis function, decision tree, and linear discriminant analysis. In this work, we also evaluated the importance of each feature by starting with the single top-ranked feature in each dataset and incrementally adding another feature with the next highest probability for iterative classification of glycemic control until all features were exhausted. Figure 3 shows that the top-ranked feature in the SAP dataset was prior-day TAR = 0% which is associated with good glycemic control while the top-ranked feature in the HCL dataset is prior-day TAR between 36 and 100% which is associated with poor glycemic control. The scikit-learn machine learning library in Python was used for implementation^81^. For performance evaluation, we used established metrics including F1 score, Recall, and Precision. However, we also reported the balanced accuracy^60^ to account for inherent skewness and class imbalance in the datasets as shown in Fig. 2. As a final validation step, we compared top-ranked features across both the SAP and HCL datasets, to assess consistency in our findings and identify the most impactful digital biomarkers for daily glycemic control.

### Reporting summary

Further information on research design is available in the Nature Research Reporting Summary linked to this article.

## Supplementary information

Supplementary material

Reporting Summary

## Data Availability

The data analyzed in this study is available through the Tidepool Big Data Donation Project^68^ but restrictions may apply. While data sharing agreements prohibit the authors from making the dataset publicly available, confidential access may be granted to those who meet specific criteria, pending permission from Tidepool. Access can be requested through the corresponding author, T.P.

## Code availability

The code generated for this study is available upon request at the Augmented Health Lab at Dartmouth College (www.ah-lab.cs.dartmouth.edu/).
