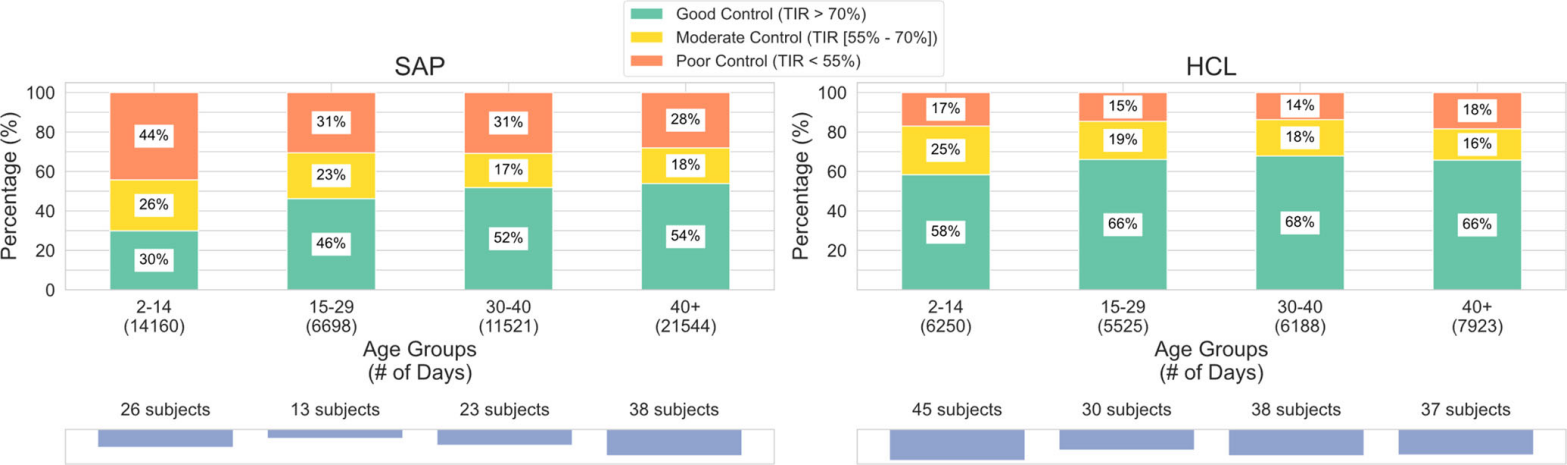# Author Correction: A computational framework for discovering digital biomarkers of glycemic control

**DOI:** 10.1038/s41746-022-00686-7

**Published:** 2022-09-06

**Authors:** Abigail Bartolome, Temiloluwa Prioleau

**Affiliations:** Dartmouth College, Computer Science, Hanover, NH 03755 USA

**Keywords:** Biomedical engineering, Type 1 diabetes

Correction to: *npj Digital Medicine* 10.1038/s41746-022-00656-z, published online 08 August 2022

Fig. 5 “SAP” was duplicate image of “HCL”; the figure should have appeared as shown below. The original article has been corrected.