# Supplementary material for: A computational framework for discovering digital biomarkers of glycemic control
Source: NPJ Digit Med. 2022 Aug 8;5:111. doi: 10.1038/s41746-022-00656-z (PMC9360447; doi:10.1038/s41746-022-00656-z)
Supplement: Supplementary file 1 — Supplementary material [file 41746_2022_656_MOESM1_ESM.pdf]

Supplementary Table 1: Overview of behavioral features defined from diabetes-relevant digital devices

Digital Device	Behaviors	Bins
**CGM**	Prior Day Time in Range	Not Recorded
		1—40%
		41—60%
		61—80%
		81—100%
	Prior Day Time Above Range	Not Recorded
		1—5%
		6—35%
		36—100%
**Insulin Pump**	Basal Insulin	No Entry
		1—10 units
		10—20 units
		21—30 units
		Above 30 units
	Bolus Insulin	No Entry
		1—10 units
		10—20 units
		21—30 units
		Above 30 units
	Carb Input	No Carb Input (0—4 grams)
		5—50 grams
		51—100 grams
		101—150 grams
		151—200 grams
		201—250 grams
		More than 250 grams

Supplementary Table 2: Results from 2 additional trials of modeling behavioral features and glycemic control using supervised latent Dirichlet allocation (sLDA)

Trial		Good Control		Poor Control	
		Term	Probability	Term	Probability
1	SAP	PD-TIR (80 - 100%)	0.102	PD-TAR [36 - 100%]	0.120
		Basal (0 - 10 units]	0.091	PD-TIR [41 - 60%]	0.111
		Bolus [1 - 10 units]	0.087	Basal (> 30 units)	0.055
		PD-TAR (0%)	0.079	Carbs (50 - 100 grams]	0.055
		Bolus (10 - 20 units]	0.070	Carbs (100 - 150 grams]	0.053
		PD-TAR [1 - 5%]	0.062	Bolus (> 30 units)	0.053
		No Carb Input	0.059	Basal (10 - 20 units]	0.049
		PD-TAR [6 - 35%]	0.059	Bolus [1 - 10 units]	0.048
		PD-TIR [61 - 80%]	0.057	Bolus (10 - 20 units]	0.047
		Carbs (100 - 150 grams]	0.049	Basal (0 - 10 units]	0.046
	HCL	PD-TIR (80 - 100%)	0.107	PD-TIR [41 - 60%]	0.260
		Basal (10 - 20 units]	0.099	PD-TAR [36 - 100%]	0.254
		Bolus [1 - 10 units]	0.095	Basal (> 30 units)	0.088
		PD-TAR (0%)	0.094	Bolus [1 - 10 units]	0.071
		Basal (20 - 30 units]	0.094	Basal (20 - 30 units]	0.054
		Bolus (10 - 20 units]	0.093	Bolus (10 - 20 units]	0.053
		PD-TIR [61 - 80%]	0.087	Basal (10 - 20 units]	0.045
		PD-TAR [1 - 5%]	0.083	Bolus (20 - 30 units]	0.044
		PD-TAR [6 - 35%]	0.082	Bolus (> 30 units)	0.040
		Basal (0 - 10 units]	0.068	Basal (0 - 10 units]	0.034
2	SAP	PD-TIR (80 - 100%)	0.095	PD-TAR [36 - 100%]	0.121
		PD-TAR [6 - 35%]	0.068	PD-TIR [41 - 60%]	0.115
		PD-TIR [61 - 80%]	0.065	Bolus (> 30 units)	0.061
		PD-TAR (0%)	0.062	Basal (> 30 units)	0.057
		PD-TAR [1 - 5%]	0.062	Carbs (50 - 100 grams]	0.056
		Bolus (10 - 20 units]	0.059	Carbs (100 - 150 grams]	0.053
		Basal (0 - 10 units]	0.054	Basal (10 - 20 units]	0.053
		Basal (10 - 20 units]	0.053	Bolus (20 - 30 units]	0.050
		Basal (20 - 30 units]	0.052	Bolus (10 - 20 units]	0.046
		Bolus [1 - 10 units]	0.050	Bolus [1 - 10 units]	0.045
	HCL	PD-TIR (80 - 100%)	0.101	PD-TAR [36 - 100%]	0.262
		Basal (10 - 20 units]	0.095	PD-TIR [41 - 60%]	0.239
		Bolus (10 - 20 units]	0.094	Basal (> 30 units)	0.100
		Bolus [1 - 10 units]	0.092	Bolus [1 - 10 units]	0.063
		Basal (20 - 30 units]	0.091	Bolus (> 30 units)	0.053
		PD-TIR [61 - 80%]	0.090	Basal (20 - 30 units]	0.052
		PD-TAR [6 - 35%]	0.087	Bolus (20 - 30 units]	0.052
		PD-TAR (0%)	0.086	Bolus (10 - 20 units]	0.051
		PD-TAR [1 - 5%]	0.077	Basal (10 - 20 units]	0.040
		Bolus (20 - 30 units]	0.068	Basal (0 - 10 units]	0.026

Supplementary Table 3: Ranked Behavioral Features associated with both good and poor glycemic control in the SAP (n=100) and HCL (n=150) datasets

**SAP**	**HCL**
PD-TAR (0 or Not Recorded)	PD-TAR [36 - 100%]
PD-TAR [36 - 100%]	PD-TIR [41 - 60%]
PD-TIR [41 - 60%]	Basal (10 - 20 units]
PD-TIR (80 - 100%)	PD-TIR (80 - 100%)
Basal (0 - 10 units]	Basal (20 - 30 units]
Bolus [1 - 10 units]	Basal (30 units - INFINITY)
PD-TIR (0 or Not Recorded)	Bolus [1 - 10 units]
Bolus (30 units - INFINITY)	Bolus (10 - 20 units]
Basal (30 units - INFINITY)	PD-TAR (0 or Not Recorded)
Carbs (50 - 100 grams]	PD-TIR [61 - 80%]
Bolus (10 - 20 units]	PD-TAR [6 - 35%]
Carbs (100 - 150 grams]	PD-TAR [1 - 5%]
Basal (10 - 20 units]	Basal (0 - 10 units]
Basal (20 - 30 units]	Bolus (20 - 30 units]
Bolus (20 - 30 units]	Bolus (30 units - INFINITY)
Carbs (150 - 200 grams]	PD-TIR [1 - 40%]
PD-TAR [6 - 35%]	Bolus (Not Reported)
PD-TIR [61 - 80%]	PD - Basal (Not Reported)
No Carb Input	PD-TIR (0 or Not Recorded)
Carbs (250 grams - INFINITY)	No Carb Input
Carbs [5 - 50 grams]	
Carbs (200 - 250 grams]	
PD-TIR [1 - 40%]	
Bolus (Not Reported)	
PD-TAR [1 - 5%]	
PD - Basal (Not Reported)	

Supplementary Table 4: Breakdown of total and test blood glucose samples in the SAP and HCL dataset used in this study

	**SAP**	**HCL**
**Total Samples: Good Control**	24902	16701
**Total Samples: Poor Control**	17915	4178
**Test Samples: Good Control**	4892	3242
**Test Samples: Poor Control**	3151	835

SAP Confusion Matrix

True \ Predicted	Good Control	Poor Control
Good Control	4198	694
Poor Control	1094	2057

HCL Confusion Matrix

True \ Predicted	Good Control	Poor Control
Good Control	3012	230
Poor Control	460	375

Supplementary Figure 1 Confusion Matrix for classification of good and poor glycemic control when using a logistic regression classifier and 80/20 train/test split. An F1 score of 82.4% was obtained for the SAP population and F1 score of 89.7% for the HCL population.

Supplementary Table 5: Results obtained with 4 classifiers for predicting good versus poor glycemic control in the SAP and HCL test sets using the optimal number of features identified (i.e. the top-ranked 7 - 8 features).

Dataset	Number of Features	Classifier	F1 Score (%)	Recall (%)	Precision (%)	Balanced Accuracy (%)
**SAP**	8	Logistic Regression	82.4	85.8	79.3	75.5
		RBF-SVM	81.6	82.4	80.0	76.0
		Decision Tree	81.3	81.5	81.1	76.0
		Linear Discriminant Analysis	82.5	85.4	79.8	75.9
**HCL**	7	Logistic Regression	89.7	92.9	86.8	68.9
		RBF-SVM	89.9	92.7	87.2	69.8
		Decision Tree	89.9	92.7	87.2	69.8
		Linear Discriminant Analysis	89.2	91.7	86.9	69.0
