# Supplementary material for: A computational framework for discovering digital biomarkers of glycemic control
Source: NPJ Digit Med. 2022 Aug 8;5:111. doi: 10.1038/s41746-022-00656-z (PMC9360447; doi:10.1038/s41746-022-00656-z)
Supplement: Supplementary file 2 — Reporting Summary [file 41746_2022_656_MOESM2_ESM.pdf]

nature portfolio

Corresponding author(s): Temiloluwa Prioleau

Last updated by author(s): Apr 8, 2022

Reporting Summary

Nature Portfolio wishes to improve the reproducibility of the work that we publish. This form provides structure for consistency and transparency in reporting. For further information on Nature Portfolio policies, see our Editorial Policies and the Editorial Policy Checklist.

Statistics

For all statistical analyses, confirm that the following items are present in the figure legend, table legend, main text, or Methods section.

n/a	Confirmed	
☐	☒	The exact sample size (n) for each experimental group/condition, given as a discrete number and unit of measurement
☒	☐	A statement on whether measurements were taken from distinct samples or whether the same sample was measured repeatedly
☒	☐	The statistical test(s) used AND whether they are one- or two-sided<br>*Only common tests should be described solely by name; describe more complex techniques in the Methods section.*
☒	☐	A description of all covariates tested
☐	☒	A description of any assumptions or corrections, such as tests of normality and adjustment for multiple comparisons
☐	☒	A full description of the statistical parameters including central tendency (e.g. means) or other basic estimates (e.g. regression coefficient) AND variation (e.g. standard deviation) or associated estimates of uncertainty (e.g. confidence intervals)
☒	☐	For null hypothesis testing, the test statistic (e.g. F, t, r) with confidence intervals, effect sizes, degrees of freedom and P value noted<br>*Give P values as exact values whenever suitable.*
☒	☐	For Bayesian analysis, information on the choice of priors and Markov chain Monte Carlo settings
☒	☐	For hierarchical and complex designs, identification of the appropriate level for tests and full reporting of outcomes
☒	☐	Estimates of effect sizes (e.g. Cohen's d, Pearson's r), indicating how they were calculated

Our web collection on statistics for biologists contains articles on many of the points above.

Software and code

Policy information about availability of computer code

Data collection	No software was used.
Data analysis	All analysis was done in Python 3.10, all custom code generated for this study is available upon request at the Augmented Health Lab (www.ah-lab.cs.dartmouth.edu/).

For manuscripts utilizing custom algorithms or software that are central to the research but not yet described in published literature, software must be made available to editors and reviewers. We strongly encourage code deposition in a community repository (e.g. GitHub). See the Nature Portfolio guidelines for submitting code & software for further information.

Data

Policy information about availability of data

All manuscripts must include a data availability statement. This statement should provide the following information, where applicable:

- Accession codes, unique identifiers, or web links for publicly available datasets
- A description of any restrictions on data availability
- For clinical datasets or third party data, please ensure that the statement adheres to our policy

The data analyzed in this study is available through the Tidepool Big Data Donation project but restrictions may apply. While data sharing agreements prohibit the authors from making the dataset publicly available, confidential access may be granted to those who meet specific criteria, pending permission from Tidepool.

Field-specific reporting

Please select the one below that is the best fit for your research. If you are not sure, read the appropriate sections before making your selection.

☐ Life sciences ☒ Behavioural & social sciences ☐ Ecological, evolutionary & environmental sciences

For a reference copy of the document with all sections, see nature.com/documents/nr-reporting-summary-flat.pdf

Behavioural & social sciences study design

All studies must disclose on these points even when the disclosure is negative.

Study description	This is a retrospective data analysis study. The data used in this paper is strictly quantitative data from wearable device devices (i.e., continuous glucose monitors and insulin pumps).
Research sample	Our dataset is from the Tidepool Big Data Donation Project. It includes digital device data from 250 subjects with diabetes, primarily type 1 diabetes (age = 2–76 years, time with diabetes = 0–60 years).
Sampling strategy	No sample size calculations was performance. However, our dataset includes an average of 144,162 blood glucose samples per subject (total = 14.4 million samples across 100 subjects) in the SAP population and an average of 47,875 blood glucose samples per subject (total = 7.1 million samples across 150 subjects) in the HCL population.
Data collection	The data in this study was collected with commercially available continuous glucose monitors and insulin pumps.
Timing	The earliest start date for the data used in this study is June 2015 and the latest end date is October 2019. The specific date range of data varies across different subjects.
Data exclusions	There was no data excluded from the analysis
Non-participation	Not applicable.
Randomization	Participants were not allocated into experimental groups

Reporting for specific materials, systems and methods

We require information from authors about some types of materials, experimental systems and methods used in many studies. Here, indicate whether each material, system or method listed is relevant to your study. If you are not sure if a list item applies to your research, read the appropriate section before selecting a response.

Materials & experimental systems

n/a	Involved in the study	
☒	☐	Antibodies
☒	☐	Eukaryotic cell lines
☒	☐	Palaeontology and archaeology
☒	☐	Animals and other organisms
☐	☒	Human research participants
☒	☐	Clinical data
☒	☐	Dual use research of concern

Methods

n/a	Involved in the study	
☒	☐	ChIP-seq
☒	☐	Flow cytometry
☒	☐	MRI-based neuroimaging

Human research participants

Policy information about studies involving human research participants

Population characteristics	See above
Recruitment	Although the race and ethnic distribution of subjects in our study is unknown, there may be some underlying biases because research shows that individuals who use digital devices may not the average patient with diabetes.
Ethics oversight	Committee for Protection of Human Subjects at Dartmouth College

Note that full information on the approval of the study protocol must also be provided in the manuscript.
